# A Low Geriatric Nutritional Risk Index Is Associated with Low Muscle Volume and a Poor Prognosis among Cirrhotic Patients

**DOI:** 10.3390/medicina59122099

**Published:** 2023-11-29

**Authors:** Hirayuki Enomoto, Yukihisa Yuri, Takashi Nishimura, Naoto Ikeda, Tomoyuki Takashima, Nobuhiro Aizawa, Mamiko Okamoto, Kohei Yoshihara, Ryota Yoshioka, Shoki Kawata, Yuta Kawase, Ryota Nakano, Hideyuki Shiomi, Shinya Fukunishi, Shinichiro Shinzaki, Hiroko Iijima

**Affiliations:** Department of Gastroenterology, Hyogo Medical University, Mukogawa-cho 1-1, Nishinomiya 663-8501, Hyogo, Japan; yu-yukihisa@hyo-med.ac.jp (Y.Y.); tk-nishimura@hyo-med.ac.jp (T.N.); nikeneko@hyo-med.ac.jp (N.I.); tomo0204@hyo-med.ac.jp (T.T.); aizawa-n@hyo-med.ac.jp (N.A.); ma-okamoto@hyo-med.ac.jp (M.O.); ko-yoshihara@hyo-med.ac.jp (K.Y.); ri-yoshioka@hyo-med.ac.jp (R.Y.); sh-kawata@hyo-med.ac.jp (S.K.); yt-kawase@hyo-med.ac.jp (Y.K.); ri-nakano@hyo-med.ac.jp (R.N.); hi-shiomi@hyo-med.ac.jp (H.S.); sh-fukunishi@hyo-med.ac.jp (S.F.); sh-shinzaki@hyo-med.ac.jp (S.S.); hiroko-i@hyo-med.ac.jp (H.I.)

**Keywords:** liver cirrhosis, nutrition, GNRI, prognosis

## Abstract

*Background and Objectives*: The geriatric nutritional risk index (GNRI) is an easily calculable index that can be determined using three common clinical variables. The GNRI is suggested to be related to sarcopenia in cirrhotic patients. However, the relationship between the GNRI and the prognosis in patients with liver cirrhosis (LC) has not been reported. The aim of the present research is to study the association of the GNRI with the nutritional status, hepatic function reserve, and prognosis in patients with liver cirrhosis (LC). *Materials and Methods*: A total of 370 cirrhotic patients whose nutritional statuses were evaluated using anthropometric measurements and bioimpedance analysis were studied. The associations between the GNRI and nutritional status and the GNRI and hepatic function reserve were analyzed. We also investigated the GNRI and prognosis of patients with LC. *Results*: The median age of the enrolled patients was 66 years old, and 266 (71.9%) patients had viral hepatitis-related LC. The GNRI was shown to decrease with the progression of chronic liver disease, represented by an increased FIB-4 index and severe Child–Pugh and mALBI grades. In addition, a low GNRI (<92) was associated with severe cirrhosis-related metabolic disorders, including a low branched-chain amino acid-to-tyrosine ratio (BTR) and a low zinc value. The GNRI was positively correlated with two nutrition-related anthropometric variables (% arm circumference and % arm muscle circumference), and a low GNRI was related to a low skeletal muscle mass index (SMI) (<7.0 kg/m^2^ for men or <5.7 kg/m^2^ for women), as determined by using bioimpedance analysis. In addition, patients with a low GNRI (<92) had a poorer prognosis than those with a high GNRI (≥92) (log-rank test: *p* = 0.0161, and generalized Wilcoxon test, *p* = 0.01261). *Conclusions*: Our results suggest that a low GNRI is related to severe chronic liver disease, low muscle volume, and a poor prognosis of patients with cirrhosis.

## 1. Introduction

Patients with liver cirrhosis (LC) are frequently malnourished, and it is well known that the nutritional status affects the prognosis of patients [1,2,3]. The geriatric nutritional risk index (GNRI) is an easily calculable index that can be determined using three patient parameters: serum albumin value, actual weight, and ideal weight. The GNRI was originally reported as an index to assess the nutritional status of elderly inpatients [4]; however, it has been shown to be associated with the prognosis of patients with various diseases [5,6,7].

Regarding chronic liver diseases, the GNRI has been reported to be related to the prognosis in patients with liver cancer who have received anticancer treatments [8,9,10,11,12]. In addition, it has been suggested to be associated with sarcopenia in patients with LC [13]. These reports suggest the clinical relevance of the GNRI to several clinical aspects in cirrhotic patients, including the nutritional status, hepatic function reserve, and prognosis. However, such findings have not been adequately confirmed. In particular, the relationship between the GNRI and the prognosis of patients with LC has not been reported.

We thus herein investigated the association of the GNRI with the nutritional status and the hepatic function reserve in cirrhotic patients. We also assessed the association of the GNRI with the prognosis in patients with LC.

## 2. Patients and Methods

### 2.1. Patients and Clinial Data

In the present study, we retrospectively analyzed the patients with LC whose nutritional status was assessed in our department from February 2006 to January 2012. This study included patients who received anthropometric measurements and a bioimpedance analysis on the same day, and cases without blood data on the day of the nutritional assessment were excluded. The diagnosis of LC was based on histological findings and/or other clinical findings obtained from blood tests and/or imaging modalities including ultrasonography, computed tomography, and esophagogastroduodenoscopy [14]. The laboratory data of the patients on the day of the anthropometric measurements were analyzed.

### 2.2. Determination of the GNRI

The GNRI was determined according to the following formula: GNRI = 14.89 × serum albumin value (g/dL) + [41.7 × (actual weight/ideal weight)]. The ideal weight (kg) was determined as follows: (height [m])^2^ × 22 (kg/m^2^) [6]. The cohort was divided into two groups according to the cutoff value described in a previous report [4]; namely, patients with a low GNRI (<92) had moderate or severe nutritional risk, whereas those with a high GNRI (≥92) had low or no nutritional risk [4].

### 2.3. Determination of Data Related to the Hepatic Function Reserve and Liver Fibrosis

In the present study, we evaluated the association of the GNRI with the degree of liver fibrosis and hepatic function reserve. The degree of liver fibrosis was assessed using the FIB-4 index [15], which was determined using the following calculation [15]: FIB-4 index—age (years) × AST (U/L)/(platelet count [10^9^/L] × (ALT [U/l]^1/2^). With regard to the hepatic function reserve, we classified the enrolled patients into three groups according to the Child–Pugh grade (grades A, B, and C). In addition, we calculated the albumin–bilirubin (ALBI) score [16] and classified the patients into four groups according to the modified ALBI (mALBI) grade (grades 1, 2a, 2b, and 3) [17]. The ALBI score and mALBI grade were calculated as follows [16,17,18]:

ALBI score = (log_10_ bilirubin (μmol/L) × 0.66) − (serum albumin (g/L) × 0.085)

mALBI grade: Grade 1 (ALBI score ≤ −2.60); Grade 2a (ALBI score > −2.60 to −2.27); Grade 2b (ALBI score > −2.27 to −1.39); Grade 3 (ALBI score > −1.39).

In the present study, we also assessed the association of the GNRI with two cirrhosis-related metabolic variables: the branched-chain amino acid (BCAA)-to-tyrosine ratio (BTR) [19,20] and zinc concentration [21,22]. The BTR correlates well with Fisher’s ratio, and its decrease reflects an amino acid imbalance [19,20], while a low zinc value is related to various clinical disorders, including hyperammonemia [21,22].

### 2.4. The Evaluation of Anthropometric Data Representing Energy Malnutrition and Muscle Volume Loss

Two anthropometry-based nutritional parameters, arm circumference (AC) and arm muscle circumference (AMC), were assessed in the present study. A nominated nutritionist manually measured the values of AC (cm) and triceps skinfold thickness (TSF) (cm) [23], and the values of AMC were determined according to the following formula:

AMC (cm) = AC (cm) − TSF × π (cm).

The values of %AC and %AMC were calculated according to the standard values of AC and AMA in Japanese individuals [24]. Decreased %AC and %AMC values were considered to represent energy malnutrition and muscle volume loss, respectively [23]. 

### 2.5. The Body Composition Analysis and Determination of the Skeletal Muscle Mass Index (SMI)

In the present study, using bioimpedance analysis (BIA; InBody720^®^, Inbody Japan, Tokyo, Japan), we assessed the body composition of the patients. The SMI was determined using the following formula:

SMI (kg/m^2^) = (total appendicular muscle mass [kg])/(height [m])^2^.

Cases with a low skeletal muscle volume were determined according to the cutoff value (SMI < 7.0 kg/m^2^ for men or SMI < 5.7 kg/m^2^ for women) in the Japanese guidelines for sarcopenia in liver disease [5].

### 2.6. Statistical Analyses

Regarding continuous variables, the relationships between the two parameters were assessed using Spearman’s correlation coefficient. Statistical differences between two groups were evaluated using Wilcoxon’s rank-sum test, and the Kruskal–Wallis test and Steel–Dwass test were used for the comparisons among three or more groups. Regarding the assessment of the prognoses, Kaplan–Meier survival curves were generated, and the log-rank test and generalized Wilcoxon’s test were used. *p*-values less than 0.05 were considered as statistically significant.

## 3. Results

### 3.1. Basic Characteristics of the Patients with LC

In the present study, 370 patients with cirrhosis who underwent anthropometric measurement were studied. The basic clinical characteristics of the patients are shown in Table 1. The enrolled cases comprised 219 men (59.1%) and 151 women (40.9%). The median age of the cohort was 66 years old, and 266 (71.9%) patients had viral hepatitis-related LC. A total of 169 patients (45.6%) had decompensated LC with Child–Pugh grades B or C. The median GNRI was 90.8 (interquartile range: 85.7–96.8).

### 3.2. Relationship of the GNRI with the Liver Disease Progression and Hepatic Function Reserve

**Figure 1:**
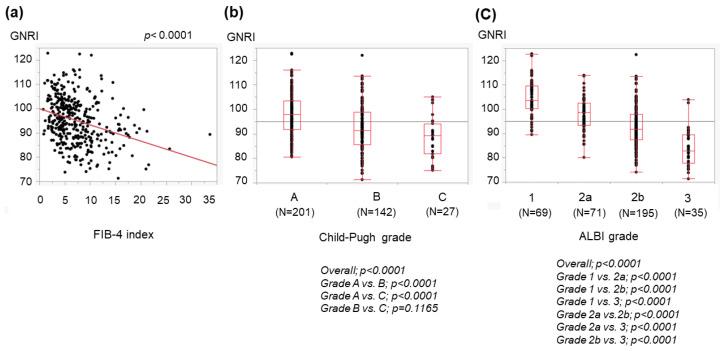
Association of the GNRI with the liver disease progression. Associations of GNRIs in cirrhotic patients with the FIB-4 index (**a**), Child–Pugh grade (**b**), and modified albumin–bilirubin (mALBI) grade (**c**). The dots show the individual data of the studied cases. The boxes indicate the interquartile ranges (IQR); namely, the top lines, the center lines and the bottom lines of the boxes express the 75th percentiles, the 50th percentiles and the 25th percentiles, respectively. The ends of the whiskers in the bar graphs extended to the data of 1.5 × IQR from the top and bottom of the boxes.

We evaluated the associations between the GNRI and the FIB-4 index, Child–Pugh grade, and mALBI grade. The GNRI decreased as the FIB-4 index increased (Figure 1a). The GNRI also decreased with increasing Child–Pugh severity (Figure 1b) and mALBI grades (Figure 1c).

### 3.3. Associations of the GNRI with LC-Related Metabolic Disorders

**Figure 2:**
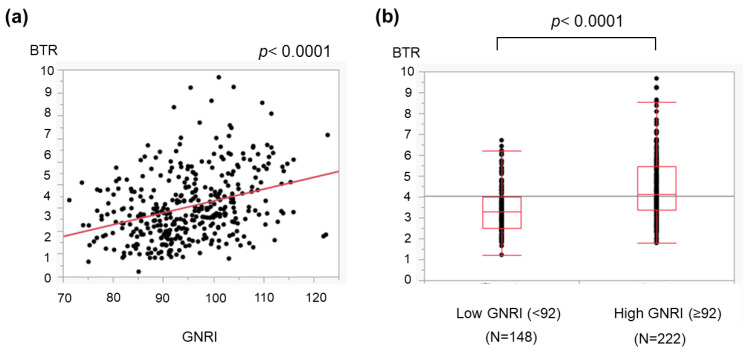
Association of the GNRI with the amino acid imbalance in patients with liver cirrhosis. The branched-chain amino acid-to-tyrosine ratio (BTR) correlates with Fisher’s ratio, and a low BTR reflects the progression of amino acid imbalance. (**a**) The GNRI was associated with the BTR. (**b**) Enrolled patients were divided into two groups according to the cutoff value used in a previous report [8]. The BTR in patients with a low GNRI (<92) was lower than that in those with a high GNRI (≥92). The dots show the individual data of the studied cases. The boxes indicate the interquartile ranges (IQR); namely, the top lines, the center lines and the bottom lines of the boxes express the 75th percentiles, the 50th percentiles and the 25th percentiles, respectively. The ends of the whiskers in the bar graphs extended to the data of 1.5 × IQR from the top and bottom of the boxes.

**Figure 3:**
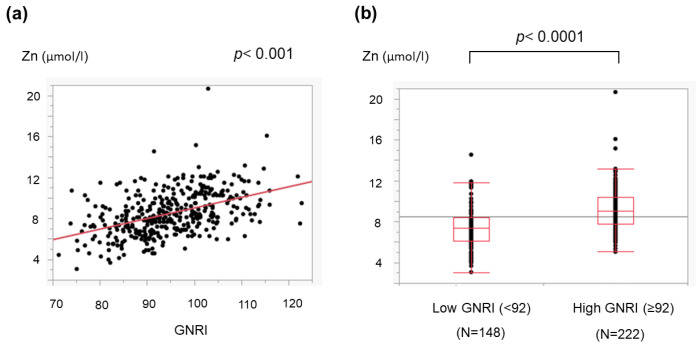
Association of the GNRI with the serum zinc value in cirrhotic patients. The zinc value correlated with the GNRI (**a**), and the zinc value in patients with a low GNRI (<92) was lower than that in those with a high GNRI (≥92) (**b**). The dots show the individual data of the studied cases. The boxes indicate the interquartile ranges (IQR); namely, the top lines, the center lines and the bottom lines of the boxes express the 75th percentiles, the 50th percentiles and the 25th percentiles, respectively. The ends of the whiskers in the bar graphs extended to the data of 1.5 × IQR from the top and bottom of the boxes.

We further assessed the relationship between the GNRI and LC-related metabolic disorders, including amino acid imbalance and low zinc levels. The GNRI was correlated with the BTR (Figure 2a). When the cohort was classified into two groups according to the cutoff value described in a previous report [4], the BTR in patients with a low GNRI (<92) was lower than that in patients with a high GNRI (≥92) (Figure 2b). Furthermore, the GNRI correlated with blood zinc concentrations (Figure 3a), and the serum zinc levels in patients with a low GNRI were significantly lower than those in patients with a high GNRI (Figure 3b).

### 3.4. Associations between the GNRI and the Anthropometric Data, the Muscle Mass Volume, and the Prognoses of LC Patients

**Figure 4:**
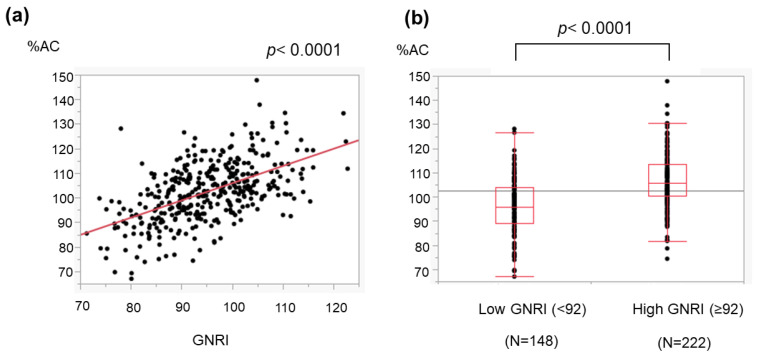
Association of the GNRI with the arm circumference (AC) in cirrhotic patients. The AC was obtained as mentioned in the ‘Section 2’. (**a**) The GNRI correlated with the %AC, which is reported to represent energy malnutrition. (**b**) The %AC in patients with a low GNRI (<92) was lower than that in those with a high GNRI (≥92). The dots show the individual data of the studied cases. The boxes indicate the interquartile ranges (IQR); namely, the top lines, the center lines and the bottom lines of the boxes express the 75th percentiles, the 50th percentiles and the 25th percentiles, respectively. The ends of the whiskers in the bar graphs extended to the data of 1.5 × IQR from the top and bottom of the boxes.

**Figure 5:**
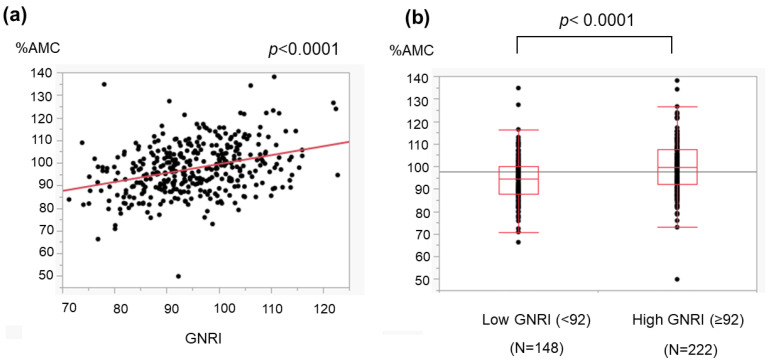
Association of the GNRI with the arm muscle circumference (AMC) in cirrhotic patients. The AMC was obtained as described in the ‘Section 2’. (**a**) The GNRI correlated with the %AMC, which is reported to represent muscle volume. (**b**) The %AMC in patients with a low GNRI (<92) was lower than that in those with a high GNRI (≥92). The dots show the individual data of the studied cases. The boxes indicate the interquartile ranges (IQR); namely, the top lines, the center lines and the bottom lines of the boxes express the 75th percentiles, the 50th percentiles and the 25th percentiles, respectively. The ends of the whiskers in the bar graphs extended to the data of 1.5 × IQR from the top and bottom of the boxes.

We evaluated the association of the GNRI with two nutrition-related anthropometric variables: %AC and %AMC. The GNRI was positively correlated with both the %AC (Figure 4) and %AMC (Figure 5).

**Figure 6:**
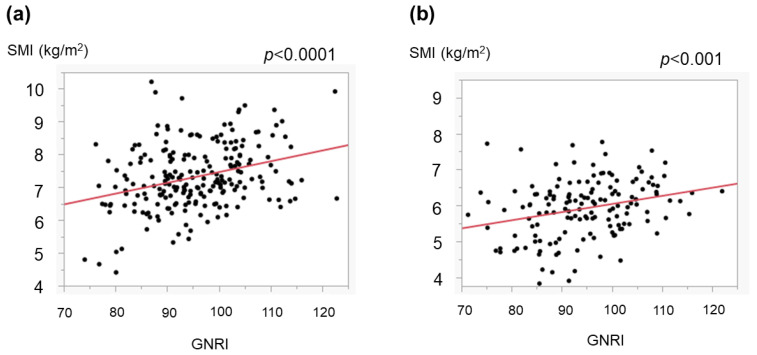
Association of the GNRI with the skeletal muscle mass index (SMI). The SMI was obtained as mentioned in the ‘Section 2’. The GNRI correlated with the SMI in both men (**a**) and women (**b**).

**Figure 7:**
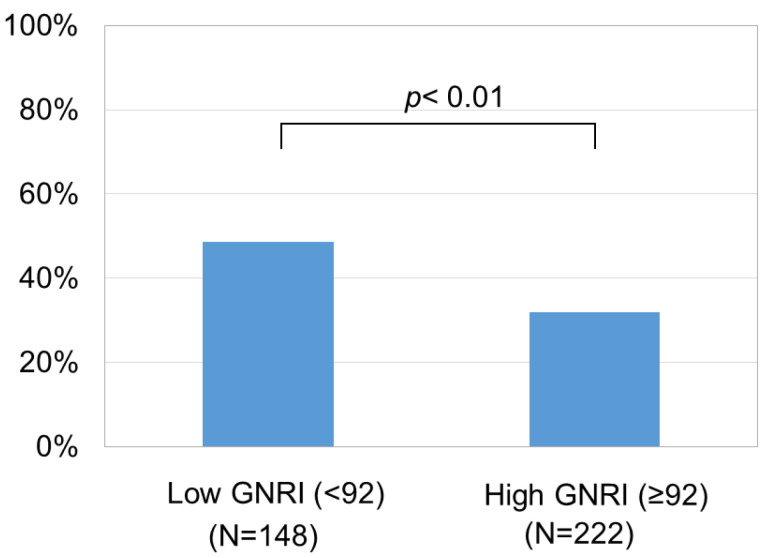
The frequency of cases with a low skeletal muscle mass index (SMI). The ratio of patients with a low SMI (an SMI < 7.0 kg/m^2^ for men and an SMI < 5.7 kg/m^2^ for women) was higher in those with a low GNRI (<92) than in those with a high GNRI (≥92).

**Figure 8:**
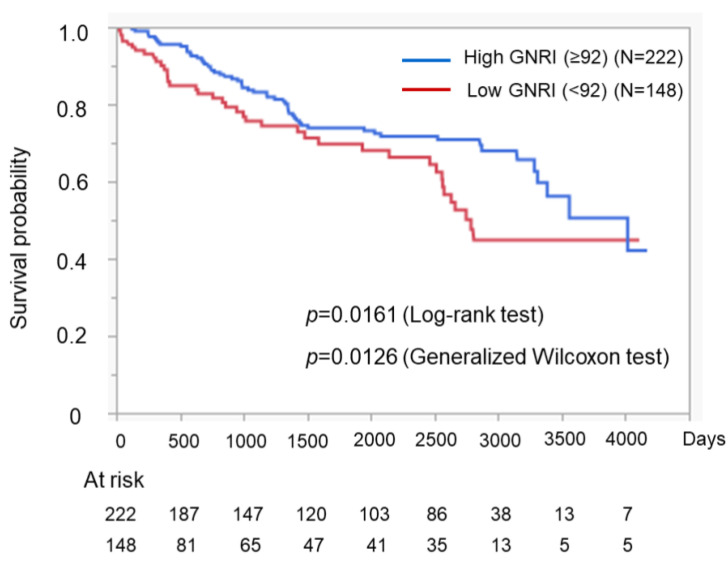
Association of the GNRI with the prognosis of cirrhotic patients. The prognoses of the patients with a high GNRI and those with a low GNRI were compared. A low GNRI was associated with an unfavorable prognosis in cirrhotic patients.

We also assessed the relationship between the GNRI and SMI. We examined the relationship separately for each sex, as the cutoff values for a low SMI differed between men and women [25]. Even so, the GNRI was correlated with the SMI in both men (Figure 6a) and women (Figure 6b). In the total cohort, the ratio of cases with a low muscle mass (low SMI) was higher in those with a low GNRI than in those with a high GNRI (Figure 7). In addition, when comparing patients with a high GNRI and those with a low GNRI, those with a low GNRI showed a poorer prognosis than those with a high GNRI (Figure 8).

## 4. Discussion

In cirrhotic patients, nutritional disorder is a frequently observed complication that can affect the prognosis. Protein energy malnutrition (PEM), which is evaluated using the serum albumin value and non-protein respiratory quotient (npRQ), is a well-known nutritional disorder in cirrhotic patients [26]. However, indirect calorimetry is required to measure the npRQ, and such a specific device is available only in limited institutions. The nutritional status can be assessed using various methodologies, such as anthropometric measurements, BIA, and dual-energy X-ray absorptiometry (DEXA) [27,28,29]; however, BIA and DEXA also require special devices. Manual anthropometric measurements require human labor, and data differences among measurers are also concerning. In our department, all anthropometric data are obtained by a designated dietician; however, such a system is not easy to prepare in every institution. The Asian guidelines for sarcopenia have introduced screening options that include a questionnaire (SARC-F and SARC-Calf) [26], and its utility has been reported [30,31,32,33]. A biomarker that can provide an objective and inexpensive nutritional assessment would therefore likely be clinically beneficial.

The GNRI can be determined using three common parameters, and we found that a low GNRI was related to severe liver disease (Figure 1) and cirrhosis-related metabolic disorders (Figure 2 and Figure 3). Recently, the GNRI was shown to be related to a low muscle volume in patients with liver cirrhosis [9] and those with hepatocellular carcinoma (HCC) [34]. Consistent with these reports, a low GNRI was associated with low nutrition-related anthropometric data (Figure 4 and Figure 5) and a decreased skeletal muscle volume (Figure 6 and Figure 7). Importantly, we showed that patients with a low GNRI score had a poor prognosis (Figure 8). The present study is the first to report the association of the GNRI in cirrhotic patients with their prognoses. Recent studies have shown that the GNRI is related to the prognosis of patients with HCC [8,9,10,11,12], and our results are consistent with these reports and support their findings. Our findings showed that a low GNRI was related to a low muscle volume and an unfavorable prognosis in cirrhotic patients. We thus believe that the GNRI can provide some important information regarding cirrhotic patients, even without any specific/expensive devices. 

The present study includes several limitations to be mentioned. First, this study was a retrospective single-center one. As a retrospective study, selection bias should be present. In addition, our institution is a core hospital, and over 40% of the enrolled patients had decompensated LC (Child–Pugh grade B or C) (Table 1). Furthermore, our hospital is located in an area with a high prevalence rate of viral hepatitis infection, and over 70% of the studied cases had viral hepatitis-related LC (Table 1). Our findings may thus depend on the specific characteristics of this cohort, so further results should be obtained in another cohort such as a nationwide survey, as recent advancements in antiviral therapies and changing lifestyles have caused an increased ratio of nonviral LC in Japan [14]. Another study in a country with different etiologies of LC and racial/ethnic backgrounds might also help address the limitations of the current study. Second, we used the cutoff value of the GNRI obtained in a previous report (GNRI < 92 and ≥92) [8,11] and showed its clinical significance. A meta-analysis reported that the association between a low GNRI and the poor survival of HCC patients was not significantly affected by the GNRI cutoff [9]. However, the optimal cutoff value should probably be determined depending on the intended use, as various cutoff values have been suggested to be related to sarcopenia in patients with chronic liver disease [9,13,34]. 

In summary, our results suggest that a low GNRI is related to severe chronic liver disease, low muscle volume, and a poor prognosis in cirrhotic patients. 

## 5. Conclusions

The GNRI was shown to decrease with the progression of chronic liver disease, and a low GNRI was associated with severe cirrhosis-related metabolic disorders, including a low BTR and low zinc value. A low GNRI was also related to low muscle volume, and patients with a low GNRI had a poorer prognosis than those with higher values. Our results suggest that a low GNRI is related to severe chronic liver disease, low muscle volume, and a poor prognosis of patients with cirrhosis. The GNRI, which can be calculated with three common variables, might help estimate the risk of sarcopenia and could be beneficial in daily practice for cirrhotic patients.

## Figures and Tables

**Table 1:** Characteristics of the enrolled patients (*N* = 370).

Age (Years)	66 (58–73)
Gender (Men/Women)	219/151
Etiology (HBV/HCV/HBV + HCV/ALD/NASH/AIH/PBC/Others)	26/238/2/40/8/16/10/30
Body Mass Index (kg/m^2^)	22.7 (20.5–25.0)
AST (IU/L)	46 (33–63)
ALT (IU/L)	34 (24–51)
GGT (IU/L)	41 (24–71)
ALP (IU/L)	319.5 (240–430)
Total bilirubin (μmol/L)	18.8 (13.7–27.4)
Albumin (g/L)	35 (31–38)
Hemoglobin (g/L)	119 (102–129)
Platelet count (×10^9^/L)	82 (61–114)
Prothrombin time (%)	75.0 (65.4–85.2)
Glucose (mmol/L)	5.61 (5.05–6.49)
Total cholesterol (mmol/L)	3.78 (3.13–4.42)
Triglyceride (mmol/L)	0.85 (0.64–1.11)
Zinc (μmol/L)	8.26 (7.04–9.95)
BTR	3.75 (2.91–4.84)
FIB-4 index	6.35 (4.18–9.41)
Child–Pugh grade (A/B/C)	201/142/27
Modified ALBI grade (1/2a/2b/3)	69/71/195/35
GNRI	90.8 (85.7–96.8)

HBV, hepatitis B virus infection; HCV, hepatitis C virus infection; ALD, alcohol-related liver disease; NASH, nonalcoholic steatohepatitis; AIH, autoimmune hepatitis; PBC, primary biliary cholangitis; AST, aspartate aminotransferase; ALT, alanine aminotransferase; GGT, gamma-glutamyl transferase; ALP, alkaline phosphatase; BTR, branched-chain amino acid (BCAA)-to-tyrosine ratio; GNRI, geriatric nutritional risk index. Data are expressed as median (interquartile range).

## Data Availability

The data shown in the present study can be provided by the corresponding author upon reasonable request.
